# Safety of rilpivirine plus nucleoside reverse-transcriptase inhibitors in HIV-infected Taiwanese with a higher prevalence of hepatitis virus infection

**DOI:** 10.7448/IAS.17.4.19580

**Published:** 2014-11-02

**Authors:** Pei-Ying Wu, Chien-Yu Cheng, Yu-Zhen Luo, Jun-Yu Zhang, Shan-Ping Yang, Shu-Hsing Cheng, Chien-Ching Hung

**Affiliations:** 1 Center for Infection Control, National Taiwan University Hospital, Taipei city, Taiwan; 2 Internal Medicine, Tao-Yuan General Hospital, Tao-Yuan city, Taiwan; 3 Internal Medicine, National Taiwan University Hospital, Taipei city, Taiwan

## Abstract

**Introduction:**

Combination antiretroviral therapy (cART) containing rilpivirine plus 2 NRTIs are effective antiretroviral (ARV) regimens for ARV-naive HIV-infected patients who had baseline plasma HIV RNA load (PVL) <5 log_10_ copies/mL and as switch therapy for those with viral suppression. In this study, we aimed to assess the short-term safety of rilpivirine-containing regimens among HIV-infected patients who initiated or switched to rilpivirine plus two NRTIs in Taiwan.

**Materials and Methods:**

Between January and June 2014, medical records of all HIV-infected patients who initiated or switched to rilpivirine plus two NRTIs, during the follow-up were reviewed to assess the tolerance and adverse effects. Using a standardized data collection form, we recorded data of PVL and CD4 count, serologies for hepatitis B and C virus (HBV and HCV, respectively), haemogram, aminotransferases, bilirubin and serum creatinine before starting rilpivirine-containing regimens at four weeks and every 12 weeks thereafter.

**Results:**

During the study period, medical records of 246 patients initiated their first ARV therapy with rilpivirine-containing regimens (n=90) or switched to rilpivirine-containing regimen from other regimens (156). Of the 246 patients, 73.4% were men who have sex with men and 9.1% and 25.6% tested positive for HBsAg and anti-HCV antibody, respectively. Baseline CD4 was 395 cells/mm^3^ (range, 2-1581) and PVL, 2.76 log_10_ copies/mL (range, <1.3>7.0 log_10_ copies/mL). As of 10 July, 23 patients (9.3%) stopped rilpivirine-containing regimens due to gastrointestinal upset (n=4), skin rash (2), depression (2), poor sleep (3), anaemia (4, all being with zidovudine/lamivudine), nail hyperpigmentation (1), presence of transmitted drug resistance (4), and elevated aminotransferase levels (1). The proportion of the patients with aminotransferases of fivefold or higher than the upper limit of normal (ULN) was 1.7% and 1.5% for AST and ALT, respectively, before starting rilpivirine-containing regimens; the respective value was 1.4% and 2.4% after 12 weeks of cART.

**Conclusions:**

Rilpivirine-containing regimens were generally well tolerated and less than 10% of the patients had to stop rilpivirine due to various reasons. Despite a higher prevalence of chronic HBV or HCV infection, rilpivirine-containing regimens did not cause significant changes of aminotransferases from baseline.

